# Associations between gut microbiota and diet composition of three arid-adapted rodent species from the Inner Mongolia grassland

**DOI:** 10.3389/fmicb.2025.1569592

**Published:** 2025-05-07

**Authors:** Muha Cha, Yunga Wu, Duhu Man, Xianfeng Yi

**Affiliations:** ^1^College of Life Sciences, Qufu Normal University, Qufu, China; ^2^Academy of Agricultural Sciences, Chifeng University, Chifeng, China; ^3^Alxa Left Banner Grassland Workstation, Bayanhot, China; ^4^College of Agriculture, Hulunbuir University, Hailar, China

**Keywords:** rodents, gut microbiota, diet, DNA metabarcoding technology, ecological adaptation strategies

## Abstract

Food habits are closely associated with the gut microbiota of herbivorous animals; however, limited knowledge exists regarding the arid-adapted rodents. This study investigates the relationship between gut microbiota and dietary composition to offer a scientific basis for comprehending the ecological adaptation strategies of grassland rodents. Cecal contents of *Spermophilus alashanicus*, *S. dauricus*, and *Meriones unguiculatus* were collected and analyzed by using 16S rRNA amplicon sequencing and DNA metabarcoding techniques to determine the structure of gut microbial communities and dietary composition. The results showed that *S. alashanicus* presented significantly higher gut microbial richness and diversity than *S. dauricus* and *M. unguiculatus*. The dominant gut bacterial genera in *S. alashanicus* and *S. dauricus* were similar, suggesting that their common genetic backgrounds might influence the colonization and symbiosis of gut microbiota. The three species consumed both plant-based and animal-based foods but differed in their dietary preferences. *S. dauricus* displayed a significantly higher diversity of animal-based food consumption compared with the other two species. Correlation analysis between diet and gut microbiota indicated that plant-based foods significantly enhanced the diversity and composition of gut microbiota. In contrast, the consumption of animal-based foods significantly decreased microbial diversity. This finding suggests a potential link between the host’s genetic background, dietary composition, and the gut microbiota.

## 1 Introduction

The intestinal microbiome exerts significant influence over the host’s metabolic processes and nutrient uptake, fostering a symbiotic relationship that benefits both parties (Tang et al., 2023; Cao et al., 2024). Through intricate interactions, gut microorganisms dynamically regulate and stabilize their community structure, thereby sustaining a robust microbial environment (Weldon et al., 2015; Lu et al., 2024). In grassland ecosystems, rodents serve dual roles as consumers and secondary producers, acting as pivotal links across various trophic levels by serving as prey for predators higher up in the food chain (Zhao et al. 2024). Their feeding habits are influenced by multiple factors, such as genetic tendencies (Suzuki et al., 2019), the nutritional quality and taste of available vegetation (Petit Bon et al., 2020), environmental conditions (Hufeldt et al., 2010), and seasonal changes (Goldberg et al., 2020). To cope with these diverse influences, rodents utilize genetic adaptations, physiological modifications, and behavioral tactics, which facilitate efficient and adaptable foraging behaviors (Brown and Kotler, 2004). These adaptive strategies not only maintain stable rodent populations within grassland ecosystems but also enable them to assume varied roles within complex ecological networks. By impacting food web dynamics, rodents significantly contribute to the stability and functionality of the ecosystem.

The structure and activities of gut microbiota are shaped by a multitude of factors, such as dietary habits, the host’s environmental conditions, and genetic makeup (Beam et al., 2021; Zhang et al., 2023; Li et al., 2016; Park and Do, 2024). Dietary intake supplies crucial nutrients for gut microbiota and modifies their composition by altering the intestinal microenvironment, including pH levels and concentrations of metabolic products (Cabana et al., 2019; Maurer et al., 2024). The host’s living conditions, like temperature, humidity, and altitude, indirectly impact gut microbiota by influencing the host’s physiological status or affecting the availability of food resources (Ley et al., 2008). Additionally, genetic elements significantly influence the composition and functionality of gut microbiota. Variations in gut morphology, immune system performance, and gene expression among hosts can substantially affect the colonization potential of specific microbes and the stability of microbial communities (Weinstein et al., 2021). DNA metabarcoding technology, an emerging tool in molecular ecology, has greatly expanded the breadth and accuracy of dietary research (Kowalczyk et al., 2019; Ter Schure et al., 2021). Utilizing high-throughput sequencing to analyze DNA fragments found in gastrointestinal contents or fecal samples, this method facilitates the swift and precise identification of species present in food sources. DNA metabarcoding has seen extensive application in studying the diets of herbivores (Kowalczyk et al., 2019; Ter Schure et al., 2021), carnivores (Wang et al., 2014; Hacker et al., 2021), and omnivores (Xiong et al., 2016; Woo et al., 2022). A comprehensive review of 155 dietary studies published from 2009 to 2020 by Ando et al. (2020) indicated that mammals are the predominant subjects of this technology. This focus is largely attributed to the ease of collecting gut samples from mammals and their significant role as key consumers in ecosystems.

In recent years, studies on the dietary behaviors of rodents have predominantly centered around their food selection tendencies, ecological niche differentiation, coexistence mechanisms among species (Sato et al., 2018; Lopes et al., 2020; Chock et al., 2022), conservation efforts for endangered species (Iwanowicz et al., 2016; Buglione et al., 2018), and the impact of environmental pollutants on their eating patterns and feeding activities (Ozaki et al., 2018). This body of research has become a critical area in rodent ecology and has laid important theoretical groundwork for comprehending adaptive strategies across different environments. The grasslands of Inner Mongolia are home to several rodent species, including *Spermophilus dauricus*, *S. alashanicus*, and *Meriones unguiculatus*. These species are recognized as carriers of plague and play a role in the indirect spread of bacterial and viral pathogens. The *S. dauricus* and the *S. alaschanicus* are closely related species. In the past, the *S. alaschanicus* was classified as a subspecies of the *S. dauricus*, but chromosomal analysis has confirmed it as a distinct species, now listed separately. The *S. alaschanicus* is mainly distributed in the western region of Inner Mongolia, while the *S. dauricus* is primarily found in the central and eastern regions of Inner Mongolia. Both are hibernating species. *S. alaschanicus* feeds on salt-alkali-tolerant plants and underground roots in extremely arid environments, with a narrow but specialized ecological niche. The *S. dauricus* feeds on the roots, stems, leaves, grass seeds, and insects of herbaceous plants, with an increase in animal-based food intake during the breeding season. The *M. unguiculatus* is a representative dominant species of desert grassland rodents, distributed in dry grasslands and agricultural areas, particularly abundant in agro-pastoral zones. It is found in the eastern, western, and central regions of Inner Mongolia, it does not hibernate and breeds year-round. After the autumn harvest, its activity in the fields increases, where it gnaws on crops and stores grains in its burrows. It mainly feeds on drought-resistant Poaceae plants, plant seeds, and crops like sorghum and millet. In wild animal gut microbiome studies, diet and host genetics are core factors shaping the microbiota. This study analyzes the food sources of three rodent species in different habitats to elucidate how food composition drives microbiota functional differentiation. By comparing the microbiota compositions of closely related species, we explore the role of genetics in shaping core microbiota. In this study, we collected and analyzed cecal samples from these three rodent species to examine their gut microbial communities using 16S rRNA amplicon sequencing. Furthermore, their dietary habits were assessed through high-throughput sequencing and DNA metabarcoding techniques. The main goal of this study is to elucidate the connection between gut microbiota and feeding behaviors in these rodents, providing valuable insights into their ecological adaptation strategies and aiding in the prevention of zoonotic disease transmission from wildlife.

## 2 Materials and methods

### 2.1 Cecal sample collection

**Figure 1:**
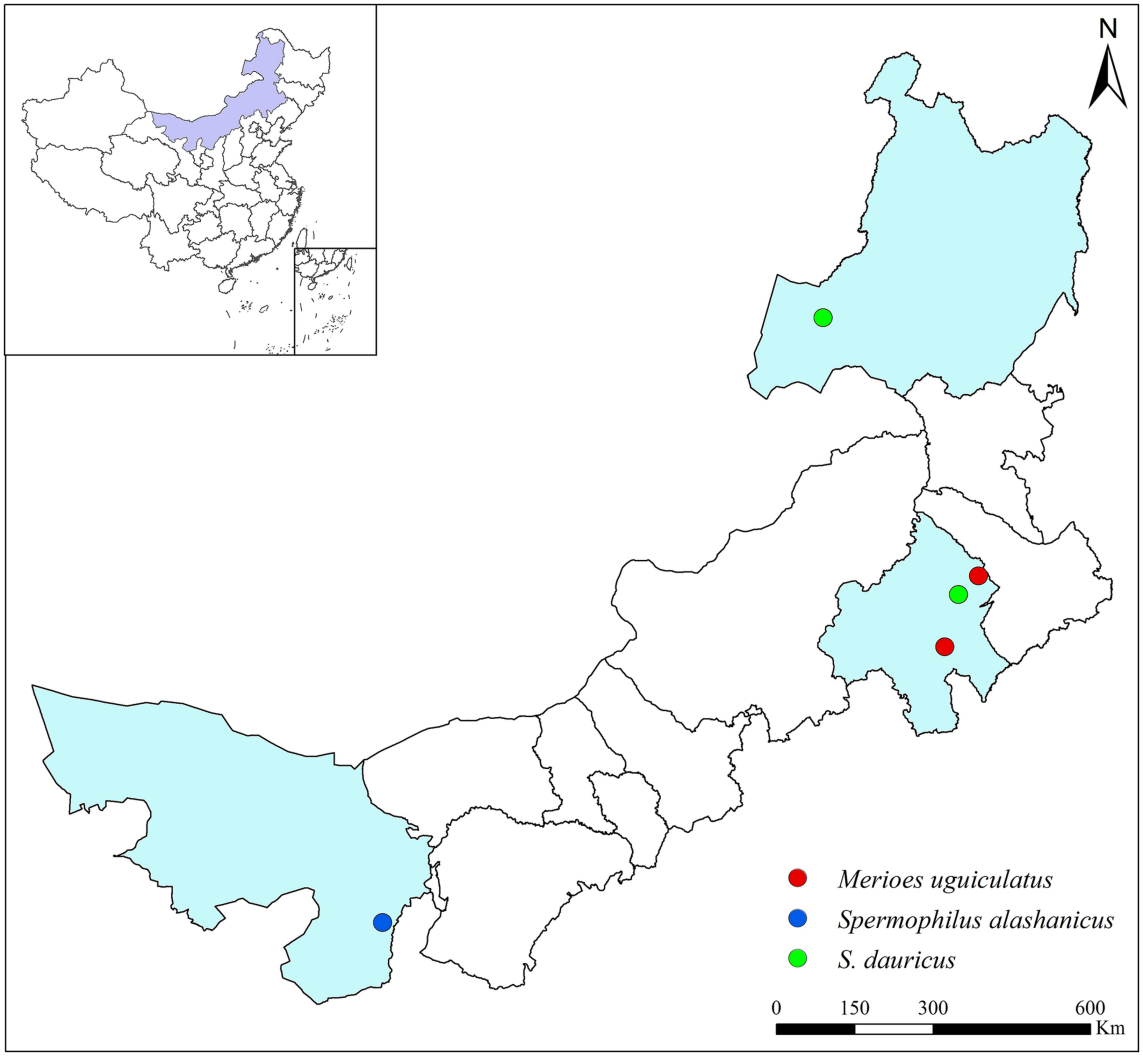
Geographical map of the specimen collection areas for the three rodent species.

In July 2023, the cecal contents of adult *S. alashanicus* (*n* = 8) were collected in Alxa League, Inner Mongolia (Supplementary Table S1). Additionally, the cecal contents of *S. dauricus* (*n* = 16) were collected in Chifeng and Hulunbuir, Inner Mongolia, while those of *M. unguiculatus* (*n* = 12) were collected in Chifeng, Inner Mongolia (Figure 1). Dissections were performed at the collection site, the contents of the cecum were placed in a freezer container and immediately frozen in liquid nitrogen. Then they were transported to the laboratory and stored in a −80°C refrigerator. The samples were sent to Shanghai Meiji Biomedical Technology Co., Ltd. and were sequenced by high-throughput Illumina PE250 sequencing platform.

### 2.2 DNA extraction and sequencing

After genomic DNA extraction, the extracted DNA was analyzed using 1% agarose gel electrophoresis. For gut microbiota analysis, primers 338F (5′-ACTCCTACGGGAGGCAGCA-3′) and 806R (5′-GGACTACHVGGGTWTCTAAT-3′) were employed for the PCR amplification of the bacterial 16S rRNA gene V3-V4 region. For plant-based food analysis, primers UniPlantF (5′-TGTGAATTGCARRATYCMG-3′) and UniPlantR (5′-CCCGHYTGAYYTGRGGTCDC-3′) were used, while primers COIF (5′-mlCOlintFGGWACWGGWTGAACWGTWTAYCCYCC-3′) and COIR (5′-jgHCO2198RTANACYTCNGGRTGNCCRAARAAYCA-3′) were utilized for the analysis of animal-based food. To determine the optimal minimum number of PCR cycles, a subset of representative samples was randomly selected for preliminary experiments. This step ensured effective amplification for most samples, producing products at suitable concentrations. PCR reactions were performed using TransStart FastPfu DNA Polymerase (TransGen AP221-02) on an ABI GeneAmp® 9700 PCR system. Each sample was amplified in triplicate, and the PCR products from the same sample were pooled. The pooled products were analyzed via 2% agarose gel electrophoresis for quality assessment, recovered using the AxyPrep DNA Gel Extraction Kit (AXYGEN), and eluted with Tris–HCl buffer. For fluorescence quantification, PCR products were measured using the QuantiFluor™-ST blue fluorescence quantification system (Promega), guided by preliminary quantification results from gel electrophoresis. Products were then combined according to sequencing requirements. Illumina adapter sequences were added to the target regions through PCR, and the resulting products were recovered by gel extraction and eluted with Tris–HCl buffer. The final products were analyzed via 2% agarose gel electrophoresis. Single-stranded DNA fragments were generated through denaturation with sodium hydroxide. During Illumina sequencing, one end of the single-stranded DNA hybridized with a complementary primer sequence fixed on the chip. Using the single strand as a template, PCR synthesis was conducted on the chip based on the fixed primer sequences. DNA clusters were generated through bridge PCR, and sequences were read via stepwise fluorescent labeling, resulting in the complete sequence of the target DNA fragments.

### 2.3 Bioinformatics analysis process

The paired-end (PE) reads obtained from Illumina sequencing were first assembled based on overlap relationships, with sequence quality controlled and filtered. The 16S rRNA genomic fragments were generated from the paired-end reads using the QIIME software (version 1.9.1). Following sample differentiation, operational taxonomic unit (OTU) clustering and taxonomic classification were performed. The OTU clustering was performed using Uparse (version 11), with a similarity threshold of 97% for clustering. OTU-based analyses enabled the evaluation of various diversity indices and the assessment of sequencing depth. Additionally, statistical analyses of community structure were conducted across multiple taxonomic levels using the taxonomic information.

### 2.4 Statistical analysis

Statistical analyses and visualizations based on the relative abundance tables of genes, species, and functions were performed using R language tools. Data clustering and abundance analyses were conducted with the vegan package in R, and the results were visualized using heatmaps. The richness and diversity of gut microbial communities were assessed using the α diversity Shannon index and Chao index. The Kruskal-Wallis H test was performed to detect significant differences between the indices of each pair of groups, and box plots were created to illustrate the differences in diversity indices among different species. Principal Coordinate Analysis (PCoA) based on the unweighted UniFrac distance matrix was utilized to evaluate differences between groups at the species, gene, and functional levels, with PCoA plots generated for visualization. In addition, non-metric multidimensional scaling (NMDS) based on based on the unweighted UniFrac metrics was applied with Analysis of similarities (ANOSIM) to analyze the difference in microbial composition among subjects and test the significance of the difference. PERMANOVA analysis, conducted using QIIME software, decomposed total variance based on Bray-Curtis distances. The non-weighted Unifrac distance was calculated by QIIME to characterize the differences in microbial communities among the samples. A linear model was applied to assess the explanatory power of different grouping factors on sample variability, and permutation tests were used to determine statistical significance. The Kruskal-Wallis rank-sum test was employed to analyze abundance differences in species and functions among groups, while the Mann–Whitney U test was applied to evaluate significant diversity differences between two groups. Spearman correlation analysis was performed to investigate the relationship between dominant gut microbiota and dietary habits. All *p*-values from statistical tests were adjusted for multiple comparisons, with significance defined as (*p* < 0.05).

## 3 Results

### 3.1 Sequencing results

**Figure 2:**
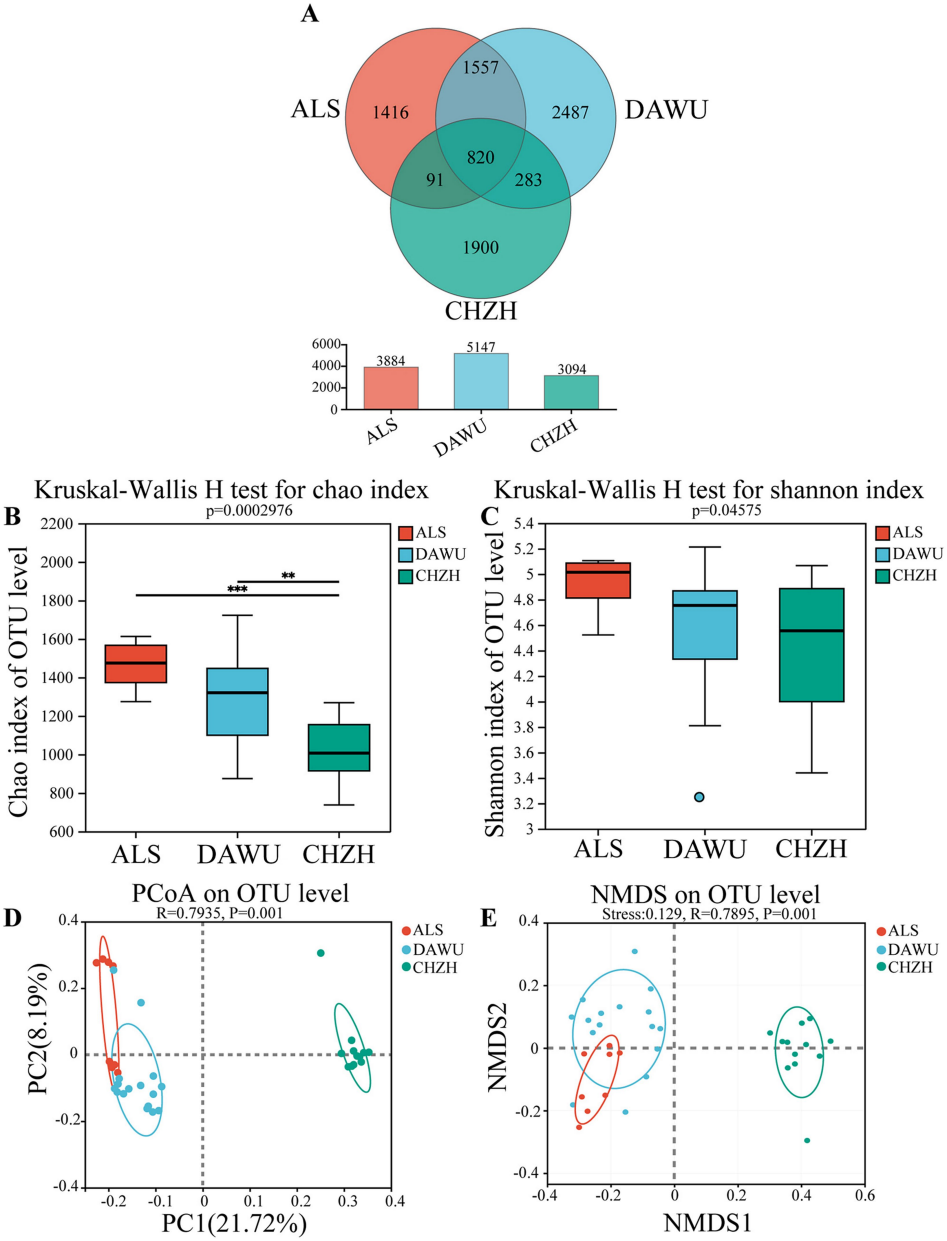
Gut microbiota diversity in the three rodent species (ALS, DAWU, and CHZH refer to *S. alashanicus*, *S. dauricus*, and *M. unguiculatus*, respectively). **(A)** Venn diagram shows shared OTUs between the gut microbiota. **(B,C)** Indicates α diversity. **(D,E)** Represent β diversity. **, and *** stand for statistically significant at *p* < 0.01, and 0.001, respectively.

A total of 36 gut microbiota samples were analyzed, comprising 8 samples from *S. alashanicus*, 16 from *S. dauricus*, and 12 from *M. unguiculatus*. Sequences were grouped into operational taxonomic units (OTUs) at 97% sequence similarity, resulting in 2,992,093 sequences and a total of 1,232,190,167 bases, with an average sequence length of 411 bp. These sequences were then clustered into 8,590 OTUs, which were taxonomically annotated into 1 domain, 1 kingdom, 17 phyla, 37 classes, 95 orders, 164 families, 333 genera, and 646 species. As sequencing depth increased, rarefaction curves for all samples approached a plateau, indicating that the sequencing depth was sufficient to capture the microbial diversity. The analysis of unique and shared OTUs among the gut microbiota of the three rodent species (Figure 2A) identified 820 shared OTUs across all species. Additionally, 2,377 OTUs were shared between *S. alashanicus* and *S. dauricus*, 1,103 between *S. dauricus* and *M. unguiculatus*, and 911 between *S. alashanicus* and *M. unguiculatus*. The highest number of shared OTUs was observed between *S. dauricus* and *S. alashanicus*.

### 3.2 Comparison of α and β diversity in the gut microbiota of the three rodent species

The gut microbiota α-diversity among the three rodent species differed significantly (Figures 2B,C). The Kruskal-Wallis H test showed significant differences in the Chao and Shannon indices among the three species (*p* < 0.05). The Chao index of *S. alashanicus* and *S. dauricus* was significantly higher than that of *Meriones unguiculatus*, while pairwise comparisons of the Shannon index revealed no significant differences among the species. To further investigate the relationship between the host and gut microbial composition, the β-diversity of the gut microbiota in *S. alashanicus*, *S. dauricus*, and *M. unguiculatus* was analyzed using principal coordinate analysis (PCoA) (Figure 2D). The PCoA based on OTU levels showed that significant differences between the groups (*R* = 0.7935, *p* = 0.001). The NMDS based on OTU levels (Figure 2E) showed that the gut bacterial samples of *S. dauricus*, *S. alaschanicus* and *M. unguiculatus* exhibited intra-group clustering but inter-group separation (Stress = 0.129; *R* = 0.7895, *p* = 0.001).

### 3.3 Analysis of gut microbiota composition

**Figure 3:**
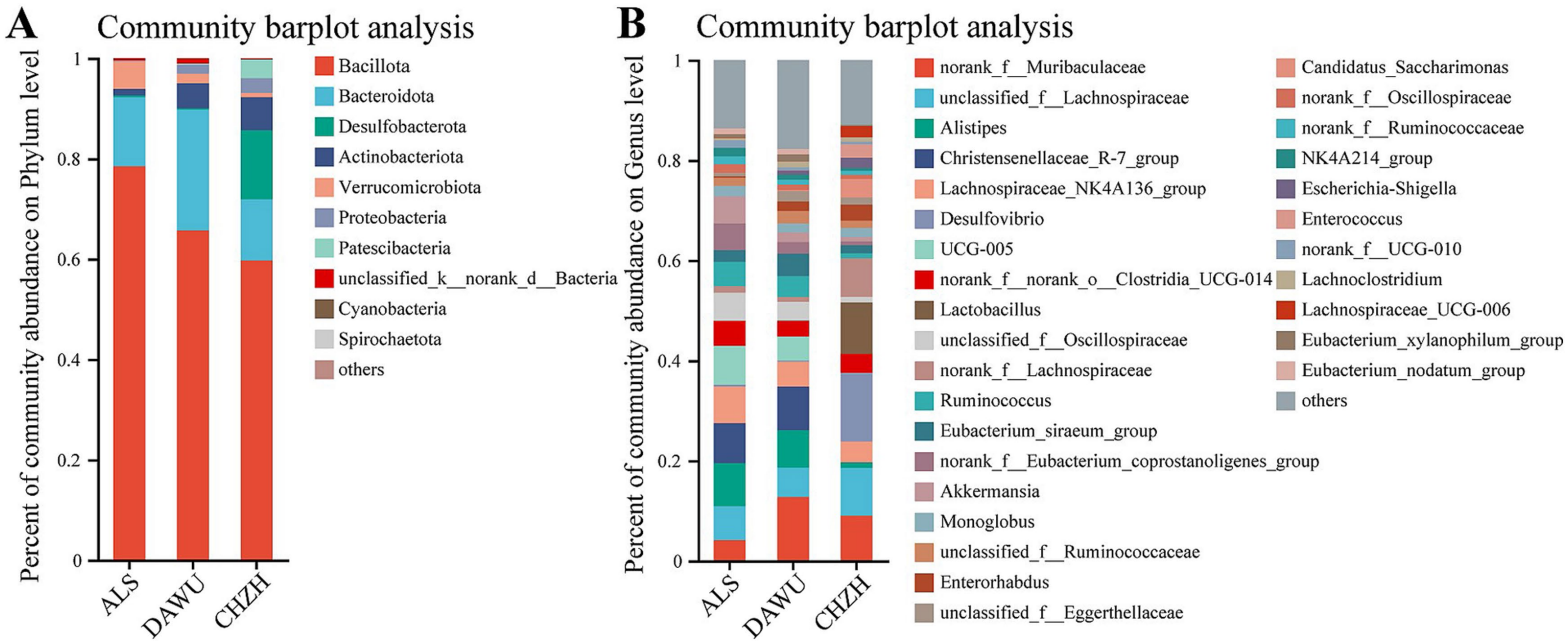
Composition of the gut microbiota at the phylum and genus level **(A,B)** (ALS, DAWU, and CHZH refer to *S. alashanicus*, *S. dauricus*, and *M. unguiculatus*, respectively).

The gut microbial community composition of *S. alashanicus*, *S. dauricus*, and *M. unguiculatus* was predominantly represented by five phyla with the highest average relative abundances: Bacillota (67.97%), Bacteroidota (16.68%), Desulfobacterota (4.79%), Actinobacteriota (4.28%), and Verrucomicrobiota (2.74%). A total of seven phyla had an average relative abundance exceeding 1%. At the genus level, the relatively abundant bacteria noted include Alistipes, Desulfovibrio, Lactobacillus. At the phylum level (Figure 3A), the dominant phyla in *S. alashanicus* were Bacillota (78.48%), Bacteroidetes (13.75%), Verrucomicrobiota (5.44%), and Actinobacteriota (1.31%). In *S. dauricus*, the primary dominant phyla were Bacillota (65.72%), Bacteroidetes (24.05%), Actinobacteriota (4.98%), and Verrucomicrobiota (1.92%). For *M. unguiculatus*, the dominant phyla included Bacillota (59.70%), Desulfobacterota (13.76%), Bacteroidota (12.22%), and Actinobacteriota (6.57%). Across all three species, Bacillota displayed the highest relative abundance, followed by Bacteroidota and Desulfobacterota. Notably, the top four dominant phyla in *S. alashanicus* and *S. dauricus* were the same, although their relative proportions differed.

At the genus level (Figure 3B), the dominant genus in *S. alashanicus* was *Alistipes* (8.61%), and in *S. dauricus*, the dominant genus was also *Alistipes* (7.47%), belonging to the phylum Bacteroidota. The primary dominant genera in *M. unguiculatus* were *Desulfovibrio* (13.63%) and *Lactobacillus* (10.29%). *Alistipes* was the shared dominant genus in both *S. alashanicus* and *S. dauricus*, while *Desulfovibrio* was the dominant genus in *M. unguiculatus*, differing from the dominant genera in the other two species.

### 3.4 The α and β diversity of plant-based and animal-based foods and the diet composition of three rodent species

**Figure 4:**
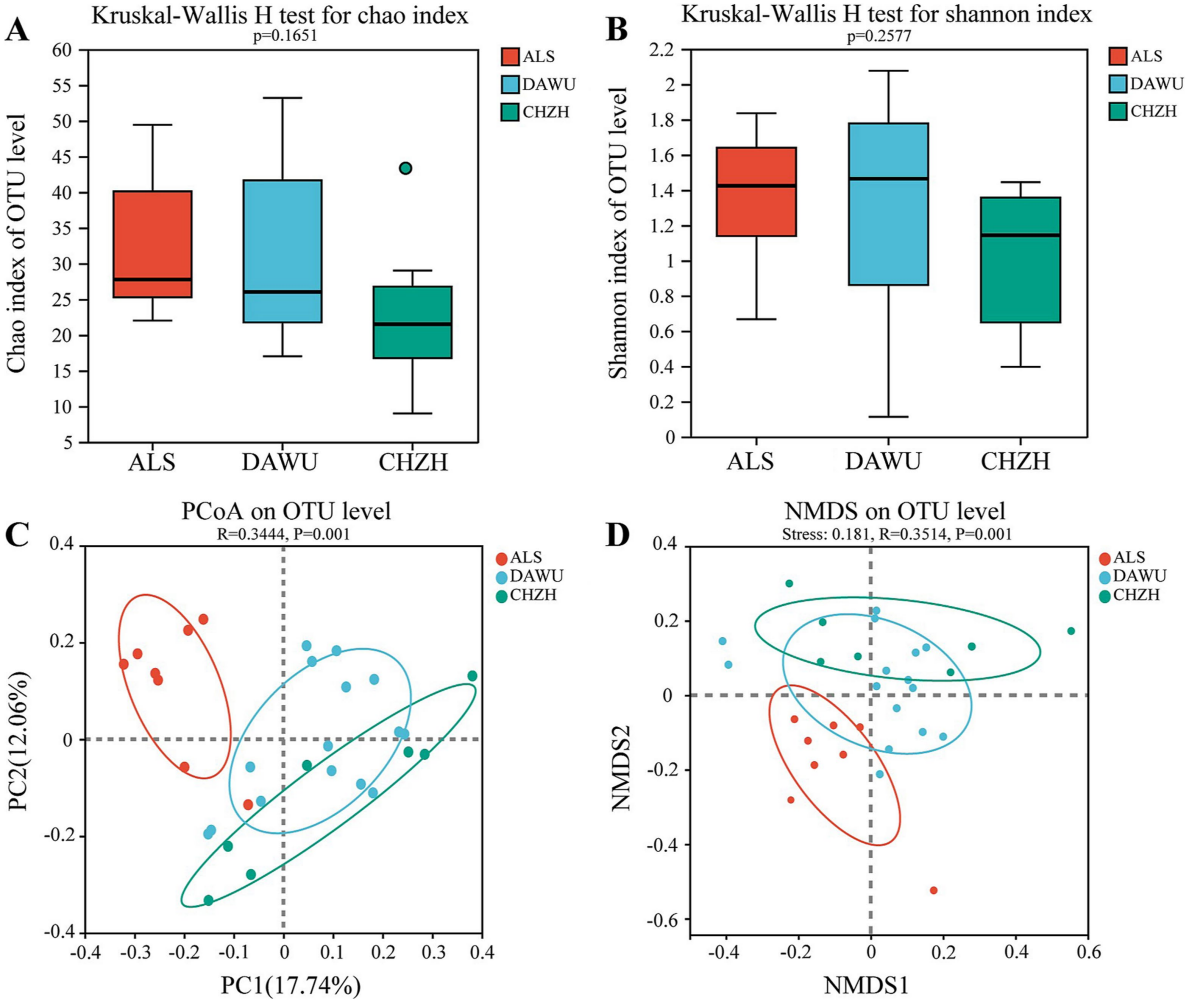
The α and β diversity index of plant-based diet composition in three rodent species (ALS, DAWU, and CHZH refer to *S. alashanicus*, *S. dauricus*, and *M. unguiculatus*, respectively). **(A,B)** Indicates α diversity. **(C,D)** Represent β diversity.

The Kruskal-Wallis H test for the α-diversity of plant-based food among the three rodent species (Figures 4A,B) revealed no significant differences in the Chao and Shannon indices (*p* > 0.05). The PCoA based on OTU levels (Figure 4C) showed that significant differences between the groups (*R* = 0.3444, *p* = 0.001). The NMDS based on OTU levels (Figure 4D) showed that the *S. dauricus*, *S. alaschanicus* and *M. unguiculatus* group samples exhibited intra-group clustering but inter-group separation (Stress = 0.181; *R* = 0.3514, *p* = 0.001).

**Figure 5:**
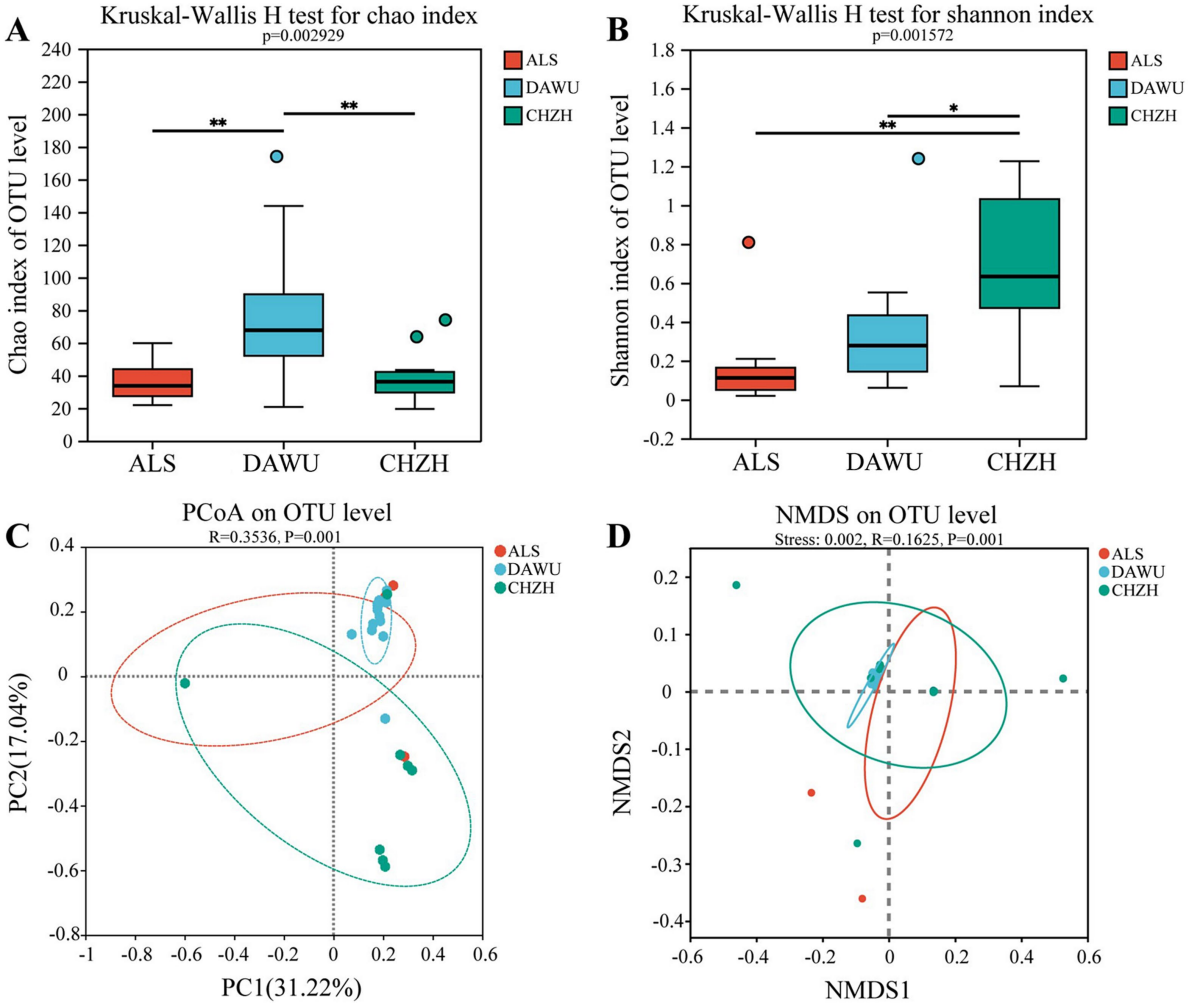
The α and β diversity index of animal-based diet composition in three rodent species (ALS, DAWU, and CHZH refer to *S. alashanicus*, *S. dauricus*, and *M. unguiculatus*, respectively). **(A,B)** Indicates α diversity. **(C,D)** Represent β diversity. *, and ** stand for statistically significant at *p* < 0.05, and 0.01, respectively.

For the α-diversity of animal-based food, the Kruskal-Wallis H test (Figures 5A,B) revealed that the Chao index for *S. dauricus* was significantly higher than that for *S. alashanicus* and *M. unguiculatus* (*p* < 0.05), while the Shannon index for *M. unguiculatus* was significantly higher than that for *S. dauricus* and *S. alashanicus* (*p* < 0.05). The PCoA based on OTU levels (Figure 5C) showed that significant differences between the groups (*R* = 0.3536, *p* = 0.001). The NMDS based on OTU levels (Figure 5D) showed that the *S. dauricus*, *S. alaschanicus* and *M. unguiculatus* group samples exhibited intra-group clustering but inter-group separation (Stress = 0.002; *R* = 0.1625, *p* = 0.001).

**Figure 6:**
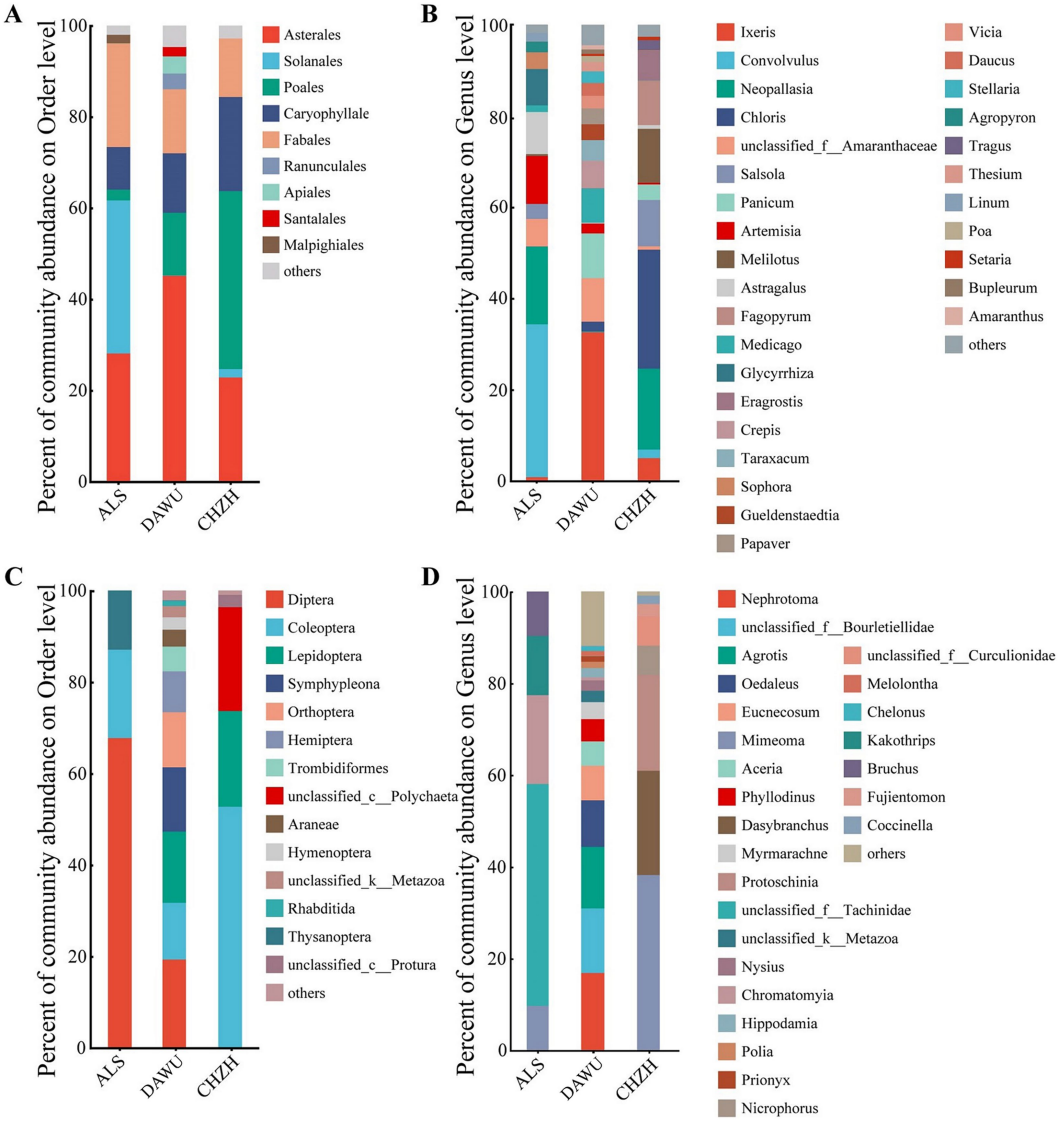
The dietary composition of the three rodent species (ALS, DAWU, and CHZH refer to *S. alashanicus*, *S. dauricus*, and *M. unguiculatus*, respectively). **(A,B)** Refer to plant-based diet composition at the order and genus level, respectively; **(C,D)** refer to animal-based diet composition at the order and genus level, respectively.

The plant-based food of the three rodent species belonged to the phylum Streptophyta and the class Magnoliopsida. At the order and genus levels, 10 orders and 27 genera exhibited relative abundances greater than 1% (Figures 6A,B). At the order level, the most abundant taxa included Asterales, Poales, Fabales, and Solanales. Among these, the plant-based food of *S. alashanicus* was dominated by Solanales (33.52%), *S. dauricus* by Asterales (43.13%), and *M. unguiculatus* by Poales (39.03%). At the genus level, the most abundant taxa in *S. alashanicus* were *Convolvulus* (33.52%), *Neopallasia* (17.08%), and *Artemisia* (10.52%). In *S. dauricus*, the most abundant taxa were *Ixeris* (32.57%), unclassified_f__Amaranthaceae (9.52%), and *Panicum* (9.81%). In *M. unguiculatus*, the most abundant genera were *Chloris* (26.08%), *Neopallasia* (17.78%), and *Melilotus* (11.86%).

The animal-based food of *S. alashanicus*, *S. dauricus*, and *M. unguiculatus* was predominantly composed of invertebrates from the phylum Arthropoda (Figures 6C,D). The diet of *S. alashanicus* consisted entirely of arthropods (Arthropoda, 100%), while the diet of *S. dauricus* included Arthropoda (94.70%), unclassified_k__Metazoa (2.48%), and Cnidaria (1.69%). For *M. unguiculatus*, the animal-based food consisted of Arthropoda (77.27%) and Annelida (22.72%). At the order level, the animal-based food of all three species was dominated by Diptera, Lepidoptera, Coleoptera, Thysanoptera, Collembola, and Tubicola. At the genus level, the most abundant taxa in *S. alashanicus* were unclassified_f__Tachinidae (48.39%), *Chromatomyia* (19.35%), and *Kakothrips* (12.90%). In *S. dauricus*, the dominant genera were *Nephrotoma* (16.80%), unclassified_f__Bourletiellidae (14.09%), and *Agrotis* (13.42%). For *M. unguiculatus*, the most abundant taxa were *Mimeoma* (38.18%), *Dasybranchus* (22.72%), and *Protoschinia* (20.91%).

### 3.5 The association between diet and gut microbiota

**Figure 7:**
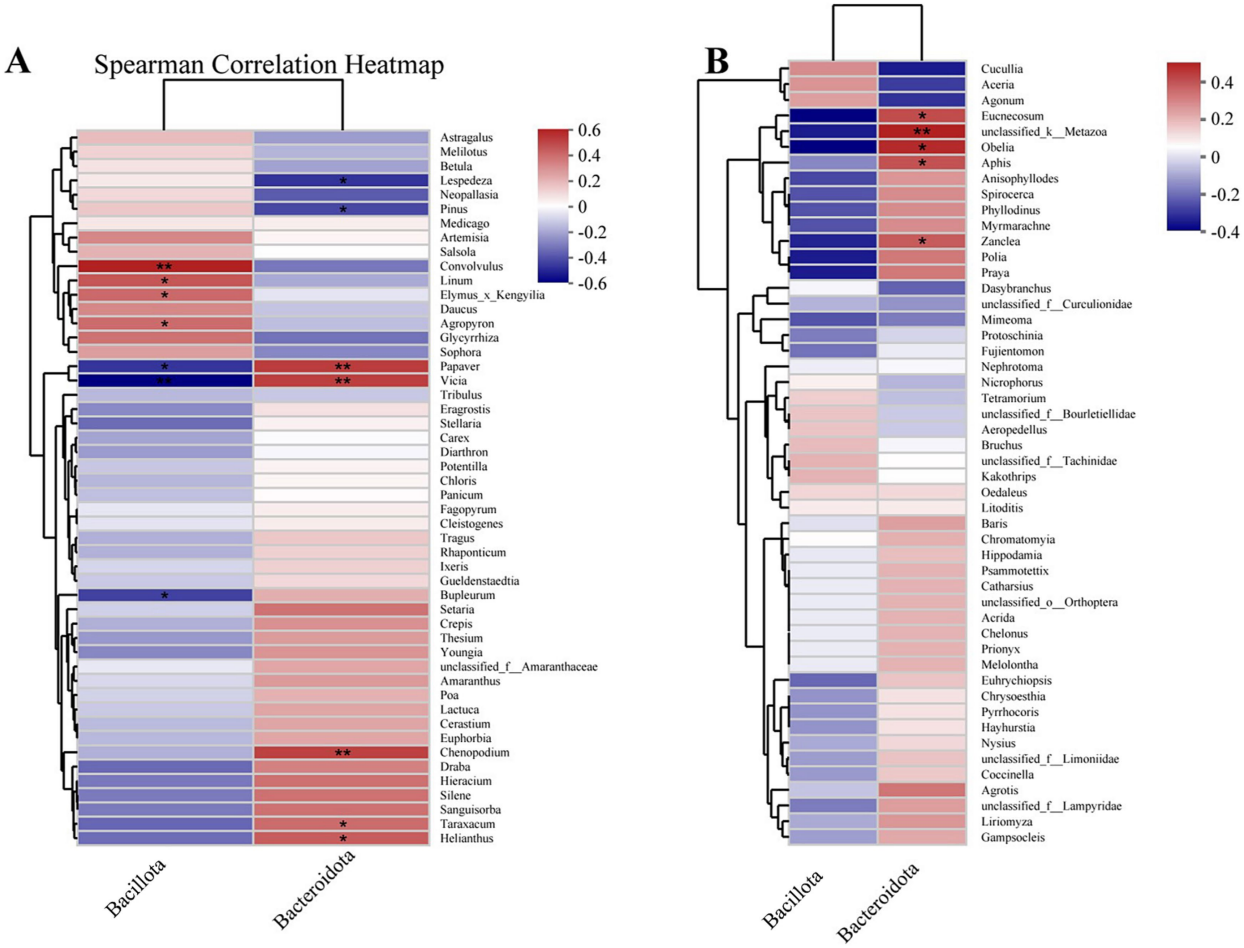
Associations between gut microbiota and diet composition of three rodent specie. **(A)** Correlation between the abundance of microbial phylum (Bacillota and Bacteroidota) and plant-based diet genera. **(B)** Correlation between the abundance of microbial phylum (Bacillota and Bacteroidota) and animal-based diet genera. *, and ** stand for statistically significant at *p* < 0.05, and 0.01, respectively.

The spearman correlation analysis of plant-based food composition and Bacillota and Bacteroidota (Figure 7A) revealed that *Convolvulus*, *Linum*, *Elymus*, and *Agropyron* were significantly positively correlated with Bacillota, whereas *Vicia*, *Papaver*, and *Bupleurum* showed significant negative correlations. For Bacteroidota, significant positive correlations were observed with *Taraxacum*, *Papaver*, *Vicia*, *Helianthus*, and *Chenopodium*, while *Lespedeza*, and *Pinus* displayed significant negative correlations. The spearman correlation analysis of animal-based food composition and Bacillota and Bacteroidota (Figure 7B) indicated that *Eucnecosum*, unclassified_k__Metazoa, *Obelia*, *Aphis*, and *Zanclea* were significantly correlated with Bacteroidota, while no significant correlations were observed with Bacillota.

**Figure 8:**
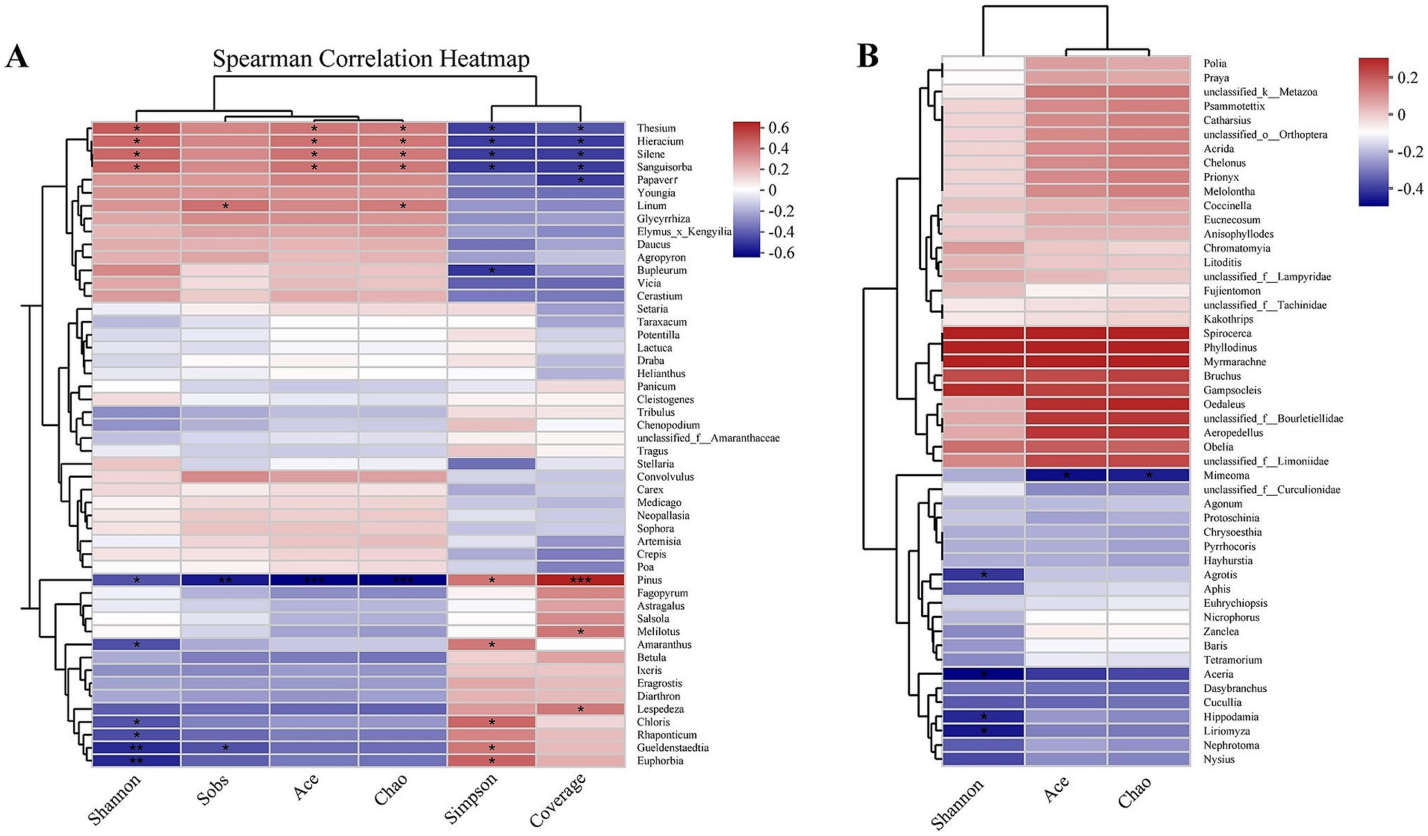
Associations between gut microbiota α diversity and diet composition of three rodent species. **(A)** Correlation between the gut microbiota α diversity and plant-based diet genera. **(B)** Correlation between the gut microbiota α diversity and animal-based diet genera. *, **, *** stand for statistically significant at *p* < 0.05, 0.01, and 0.001, respectively.

The spearman correlation analysis of plant-based food composition and gut microbiota diversity (Figure 8A) demonstrated that *Thesium*, *Silene*, *Sanguisorba*, and *Hieracium* were significantly positively correlated with the Shannon, Ace, and Chao indices, but significantly negatively correlated with the Simpson and Coverage indices. In contrast, *Chloris*, *Gueldenstaedtia*, and *Euphorbia* showed significant negative correlations with the Shannon index, while exhibiting significant positive correlations with the Simpson index. The spearman correlation analysis of animal-based food composition and gut microbiota diversity (Figure 8B) revealed that *Agrotis*, *Aceria*, *Hippodamia*, and *Liriomyza* were significantly negatively correlated with the Shannon index. Meanwhile, *Mimeoma* showed significant negative correlations with the Ace and Chao indices.

## 4 Discussion

This study examined the cecal contents of three rodent species from the Inner Mongolia grasslands: *S. alashanicus*, *S. dauricus*, and *M. unguiculatus*. Through 16S rRNA amplicon sequencing, it was found that the gut microbiota of *S. alashanicus* and *S. dauricus* was primarily composed of the phyla Bacillota, Bacteroidetes, Thermodesulfobacteria, and Actinobacteria. In contrast, the gut microbiota of *M. unguiculatus* was mainly characterized by the presence of Bacillota, Verrucomicrobia, Bacteroidetes, and Actinobacteria. Ley et al. (2008) conducted an analysis of the gut microbiota across 60 mammalian species, identifying the six most prevalent phyla as Bacillota, Bacteroidetes, Proteobacteria, Actinobacteria, Verrucomicrobia, and Fusobacteria. Similarly, studies on the gut microbiota of *Marmota himalayana* and *Ictidomys tridecemlineatus* revealed distinct compositions. The gut microbiota of *Marmota himalayana* was predominantly dominated by Proteobacteria, Bacillota, Bacteroidetes, and Actinobacteria (Xu et al., 2021), while that of *Ictidomys tridecemlineatus* was mainly comprised of Bacteroidetes, Bacillota, and Verrucomicrobia (Carey et al., 2013). The gut microbiota composition observed in this study for the three rodent species shows some similarities with previous findings, although notable interspecies differences were evident. Specifically, Bacillota exhibited the highest relative abundance across all three species. The core gut microbiota of rodents is jointly regulated by the host’s genetic background and external environmental factors, with diet serving as a key environmental variable. By providing substrate selection pressure and regulating metabolic products, diet plays a decisive role in the structural stability and functional adaptability of microbial communities (Fu et al., 2021; Weinstein et al., 2021).

These findings underscore the significance of integrating both genetic and environmental factors when investigating the gut microbiota of various rodent species. Alpha diversity serves as an indicator of microbial richness and diversity within a community. Specifically, the Chao index assess microbial richness, while the Shannon index measures diversity. Among the studied species, *S. alashanicus* and *S. dauricus* exhibits significantly higher Chao indices compared to *M. unguiculatus*. The habitats and dietary patterns of *S. alashanicus* and *S. dauricus* appear to foster richer and more diverse gut microbiota. Both habitat-specific food resources and host genetics play crucial roles in shaping gut microbiota composition (Sanders et al., 2014; Moran et al., 2019), with specific dietary nutrients promoting the enrichment of certain bacterial groups. Notably, *S. alashanicus*, which inhabit the arid desert regions of Inner Mongolia with relatively limited plant resources, exhibit higher alpha diversity indices compared to the other two species. This increased gut microbial diversity likely enhances the host’s adaptability to environmental changes. Moreover, the gut microbiota of wild mice differs substantially from that of laboratory mice, with wild mice harboring microbial communities that promote host health and improve disease resistance (Rosshart et al., 2017). Animals with specialized diets host unique microbial profiles in their guts, facilitating adaptation to specific environments and dietary conditions. In summary, these results highlight the interplay between environmental factors and host genetics in shaping gut microbial communities, emphasizing the importance of considering both elements in ecological and evolutionary studies of rodents.

Despite being collected from sites 1,500 km apart, *S. alashanicus* and *S. dauricus* both belong to the *Sciuridae* family’s *Spermophilus* genus. Their phylogenetic relationship leads to notable similarities in their gut microbiota composition, with *Alistipes* being dominant genera in both species. This contrasts sharply with *M. unguiculatus*, where Desulfovibrio is the dominant genus. The shared gut microbial characteristics between *S. alashanicus* and *S. dauricus*, despite the considerable geographical distance, suggest that host genetics play a pivotal role in shaping gut microbiota (Amato et al., 2019). From the standpoint of microbial colonization, the genetic background of the host may influence the selection of microbes during the early stages of gut colonization. Specific physiological traits of the gut can favor the adhesion and proliferation of certain microbes. Over time, the host’s genetically determined immune system facilitates the recognition and response to gut microbes, supporting the stable presence and functionality of beneficial microorganisms. For example, *Alistipes* are known for their ability to continuously metabolize dietary fiber, produce short-chain fatty acids, and modulate immunity (Parker et al., 2020; Ma et al., 2022). In terms of long-term functional co-evolution, these two closely related *Spermophilus* species have likely undergone co-evolution between host digestive system-related genes and gut microbiota, particularly concerning digestive functions. This co-evolution enables certain microbes to assist the host in enhancing nutrient acquisition efficiency, thereby occupying a dominant position in the gut. This strongly supports the critical influence of host genetic factors on gut microbiota composition, colonization, and the maintenance of symbiotic relationships. These findings provide important evidence for a deeper understanding of the mutual relationship between hosts and their gut microbial communities. They highlight how host genetics and environmental factors jointly shape the gut microbiota, influencing both its composition and function. This interplay underscores the importance of considering both genetic and ecological factors when studying the gut microbiota of different rodent species.

The dietary composition of three rodent species—*S. alashanicus*, *S. dauricus*, and *M. unguiculatus*—was analyzed using DNA metabarcoding technology. The results indicated that all three species consume both plant and animal matter. At the order level, the plant-based diet of *S. alashanicus* was predominantly composed of Solanales, while *S. dauricus* favored Asterales, and *M. unguiculatus* preferred Poales. For animal-based components, *S. alashanicus* primarily consumed arthropods, *S. dauricus* included unclassified arthropods within k__Metazoa and members of the phylum Nematoda, and *M. unguiculatus* fed on arthropods and annelids. Compared to traditional dietary analysis methods, DNA metabarcoding significantly enhances species resolution and accuracy in identifying animal-based food components, while also providing a more comprehensive analysis of plant-based dietary elements. Most plant species identified in the rodents’ diets were present across their sampling sites, further validating the reliability of the study’s findings. DNA metabarcoding has emerged as a crucial tool for analyzing dietary composition in animals (Buglione et al., 2018; Zhang et al., 2022; Pinho et al., 2022). Traditional methods often underestimate dietary diversity. For instance, Soininen et al. (2014) used high-throughput sequencing of the chloroplast gene trnL to analyze the stomach contents of *Myodes rufocanus* and *Microtus oeconomus*. Their findings revealed that traditional microscopic methods identified mosses as the primary food source, whereas metabarcoding uncovered a much more diverse diet. Similarly, Iwanowicz et al. (2016) employed DNA metabarcoding to investigate the diet of the endangered Pacific pocket mouse. Due to its nocturnal behavior, the dietary characteristics of this species were previously poorly understood. By sequencing DNA from fecal samples, they identified multiple plant genera within individual samples. Regarding animal-based food composition, the Chao index for *S. dauricus* was significantly higher than those for *S. alashanicus* and *M. unguiculatus*, indicating greater diversity in the animal-based diet of *S. dauricus*. This diversity is likely influenced by its habitat, foraging behavior, or population structure (Zhong et al., 2016). In contrast, the Coverage indices for *M. unguiculatus* and *S. alashanicus* were significantly higher than those for *S. dauricus*, suggesting more thorough utilization of animal-based food resources. No significant differences were observed in the plant-based food diversity indices among the three species, implying similar breadth and evenness in their use of plant-based resources. Despite being collected from different habitats, these three rodent species have evolved similar digestive physiological mechanisms and foraging strategies over time, leading to convergent patterns in their utilization of plant-based foods (Kartzinel et al., 2019). This convergence highlights the adaptability and flexibility of these species in response to varying environmental conditions.

The *S. alaschanicus* relies on salt-alkali tolerant plants, such as *Convolvulus* (Convolvulaceae) and *Artemisia* (Asteraceae), in extremely arid environments. High salt, high fiber, and secondary metabolites promote the growth of Bacillota, a phylum with strong tolerance, and functional bacterial genera like Alistipes. Alistipes breaks down complex carbohydrates to support host energy acquisition; synthesizes SCFAs to regulate metabolism and immunity; degrades toxins and adapts to stress, enhancing the host’s environmental adaptability. The intestinal microbiota composition of the *S. dauricus* is highly synchronized with its omnivorous dietary habits. Its plant-based diet primarily consists of Asteraceae (e.g., Ixeris), which is rich in soluble fiber and may promote the proliferation of Bacillota. This fermentation generates short-chain fatty acids (SCFAs), enhancing the host’s energy metabolism efficiency. The animal-based diet, primarily consisting of arthropods, provides protein and drives the Bacteroidetes (e.g., Bacteroides) to degrade nitrogen-containing polysaccharides. Compared to the *M. unguiculatus*, whose diet is mainly composed of Poaceae, the *S. dauricus*’s preference for Asteraceae has shaped a more efficient fiber-degrading microbiota, while animal-based food strengthens the metabolic flexibility of its gut microbiota. The intestinal microbiota composition of the *M. unguiculatus* is closely associated with its omnivorous diet, which is centered around Poales. Its plant-based food mainly consists of grasses like Chloris and Neopallasia, which are rich in complex cellulose. This may drive the enrichment of fiber-degrading microbiota (e.g., Spirillaceae or Fibrobacter), enhancing the host’s metabolic capacity for structural polysaccharides. The animal-based diet, consisting mainly of arthropods and annelids, with high intake of chitin and protein, may promote the expression of chitinase in Bacteroidetes (e.g., Mimeoma) and protein degradation functions in Proteobacteria (e.g., Protoschinia). The Poales-based diet of the *M. unguiculatus* shapes a microbiota structure better suited for high-fiber degradation.

Plant-based food is strongly and significantly associated with the gut microbiota of rodents, playing a crucial role in shaping both the abundance and diversity of gut microbial communities. The composition of different plant genera exhibits distinct correlations with specific gut microbiota phyla, underscoring the complexity and critical importance of plant-based food in regulating gut microbial ecology (Wang et al., 2022). Studies on the relationship between diet and gut microbiota in grassland rodents have also highlighted the significance of animal-based food. The composition of animal-based food showed a significant positive correlation with the abundance of Bacteroidota but no significant correlation with Bacillota, indicating its unique influence on gut microbiota structure. Furthermore, animal-based food intake was significantly negatively correlated with gut microbiota diversity indices, suggesting that its consumption reduces microbial diversity. This phenomenon may reflect an adaptive strategy, as rodents in resource-limited grassland environments likely rely on energy-dense animal-based food to supplement their diets during periods of food scarcity. Previous research on the diets of grassland rodents has predominantly focused on plant-based food, often overlooking the role of animal-based food. However, the findings of this study underscore the need to reassess the dietary composition of grassland rodents and its impact on gut microbiota. These results provide a more comprehensive understanding of the ecological adaptation strategies of grassland rodents and the intricate interactions between their diets and gut microbiota. This study offers a new perspective and key research direction for exploring the survival mechanisms and ecological roles of grassland rodents within their ecosystems. By considering both plant-based and animal-based food components, we gain deeper insights into how these rodents adapt to their environments and maintain their health through complex gut microbiota interactions. This holistic approach enhances our understanding of the ecological dynamics and survival strategies of grassland rodents.

## Data Availability

The original contributions presented in the study are included in the article/Supplementary material, further inquiries can be directed to the corresponding author.

## References

[ref1] Amato K. R. Sanders J. G. Song S. J. Nute M. Metcalf J. L. Thompson L. R.. (2019). Evolutionary trends in host physiology outweigh dietary niche in structuring primate gut microbiomes. ISME J. 13, 576–587. doi: 10.1038/s41396-018-0175-0, PMID: 29995839 PMC6461848

[ref2] Ando H. Mukai H. Komura T. Dewi T. Ando M. Isagi Y. (2020). Methodological trends and perspectives of animal dietary studies by noninvasive fecal DNA metabarcoding. Environ. DNA 2, 391–406. doi: 10.1002/edn3.117

[ref3] Beam A. Clinger E. Hao L. (2021). Effect of diet and dietary components on the composition of the gut microbiota. Nutrients 13:2795. doi: 10.3390/nu13082795, PMID: 34444955 PMC8398149

[ref4] Brown J. Kotler B. (2004). Hazardous duty pay and the foraging cost of predation. Ecol. Lett. 7, 999–1014. doi: 10.1111/j.1461-0248.2004.00661.x

[ref5] Buglione M. Maselli V. Rippa D. de Filippo G. Trapanese M. Fulgione D. (2018). A pilot study on the application of DNA metabarcoding for non-invasive diet analysis in the Italian hare. Mamm. Biol. 88, 31–42. doi: 10.1016/j.mambio.2017.10.010

[ref6] Cabana F. Clayton J. B. Nekaris K. A. I. Wirdateti W. Knights D. Seedorf H. (2019). Nutrient-based diet modifications impact on the gut microbiome of the Javan slow loris (*Nycticebus javanicus*). Sci. Rep. 9:4078. doi: 10.1038/s41598-019-40911-0, PMID: 30858577 PMC6411731

[ref9006] Cao H. Shi Y. Wang J. Niu Z. Wei L. John N. (2024). The intestinal microbiota and metabolic profiles of Strauchbufo raddei underwent adaptive changes during hibernation. Integr. Zool. 19, 612–630. doi: 10.1111/1749-4877.12749 37430430

[ref7] Carey H. V. Walters W. A. Knight R. (2013). Seasonal restructuring of the ground squirrel gut microbiota over the annual hibernation cycle. Am. J. Physiol. Regul. Integr. Comp. Physiol. 304, R33–R42. doi: 10.1152/ajpregu.00387.2012, PMID: 23152108 PMC3543654

[ref8] Chock R. Y. Shier D. M. Grether G. F. (2022). Niche partitioning in an assemblage of granivorous rodents, and the challenge of community-level conservation. Oecologia 198, 553–565. doi: 10.1007/s00442-021-05104-5, PMID: 35034220 PMC8858926

[ref10] Fu H. Zhang L. Fan C. Liu C. Li W. Cheng Q.. (2021). Environment and host species identity shape gut microbiota diversity in sympatric herbivorous mammals. Microb. Biotechnol. 14, 1300–1315. doi: 10.1111/1751-7915.13687, PMID: 33369229 PMC8313255

[ref11] Goldberg A. R. Conway C. J. Tank D. C. Andrews K. R. Gour D. S. Waits L. P. (2020). Diet of a rare herbivore based on DNA metabarcoding of feces: selection, seasonality, and survival. Ecol. Evol. 10, 7627–7643. doi: 10.1002/ece3.6488, PMID: 32760553 PMC7391308

[ref12] Hacker C. E. Jevit M. Hussain S. Muhammad G. Munkhtsog B. Munkhtsog B.. (2021). Regional comparison of snow leopard (*Panthera uncia*) diet using DNA metabarcoding. Microb. Biotechnol. 30, 797–817. doi: 10.1007/s10531-020-02062-7

[ref13] Hufeldt M. R. Nielsen D. S. Vogensen F. K. Midtvedt T. Hansen A. K. (2010). Variation in the gut microbiota of laboratory mice is related to both genetic and environmental factors. Comp. Med. 60, 336–347. doi: 10.31236/osf.io/5j7ek 21262117 PMC2958200

[ref14] Iwanowicz D. D. Vandergast A. G. Cornman R. S. Adams C. R. Kohn J. R. Fisher R. N.. (2016). Metabarcoding of fecal samples to determine herbivore diets: a case study of the endangered Pacific pocket mouse. PLoS One 11:e0165366. doi: 10.1371/journal.pone.0165366, PMID: 27851756 PMC5112926

[ref15] Kartzinel T. R. Hsing J. C. Musili P. M. Brown B. R. Pringle R. M. (2019). Covariation of diet and gut microbiome in African megafauna. Proc. Natl. Acad. Sci. 116, 23588–23593. doi: 10.1073/pnas.1905666116, PMID: 31685619 PMC6876249

[ref16] Kowalczyk R. Wójcik J. M. Taberlet P. Kamiński T. Miquel C. Valentini A.. (2019). Foraging plasticity allows a large herbivore to persist in a sheltering forest habitat: DNA metabarcoding diet analysis of the European bison. Forest Ecol. Manag. 449:117474. doi: 10.1016/j.foreco.2019.117474, PMID: 40260412

[ref17] Ley R. E. Hamady M. Lozupone C. Turnbaugh P. J. Ramey R. R. Bircher J. S.. (2008). Evolution of mammals and their gut microbes. Science 320, 1647–1651. doi: 10.1126/science.1155725, PMID: 18497261 PMC2649005

[ref18] Li H. Li T. Beasley D. E. Heděnec P. Xiao Z. Zhang S.. (2016). Diet diversity is associated with beta but not alpha diversity of pika gut microbiota. Front. Microbiol. 7:1169. doi: 10.3389/fmicb.2016.01169, PMID: 27512391 PMC4961685

[ref19] Lopes C. M. De Barba M. Boyer F. Mercier C. Galiano D. Kubiak B. B.. (2020). Ecological specialization and niche overlap of subterranean rodents inferred from DNA metabarcoding diet analysis. Mol. Ecol. 29, 3143–3153. doi: 10.1111/mec.15549, PMID: 32654383

[ref9005] Lu Y. Zhang L. Liu X. Lan Y. Wu L. Wang J. (2024). Red pandas with different diets and environments exhibit different gut microbial functional composition and capacity. Integr. Zool. 19, 662–682. doi: 10.1111/1749-4877.12813 38420673

[ref20] Ma Y. Deng X. Yang X. Wang J. Li T. Hua G.. (2022). Characteristics of bacterial microbiota in different intestinal segments of Aohan fine-wool sheep. Front. Microbiol. 13:874536. doi: 10.3389/fmicb.2022.874536, PMID: 35572716 PMC9097873

[ref9003] Maurer M. L. Goyco‐Blas J. F. Kohl K. D. (2024). Dietary tannins alter growth, behavior, and the gut microbiome of larval amphibians. Integr. Zool. 19, 585–595. doi: 10.1111/1749-4877.12758 37551631

[ref21] Moran N. A. Ochman H. Hammer T. J. (2019). Evolutionary and ecological consequences of gut microbial communities. Annu. Rev. Ecol. Evol. Syst. 50, 451–475. doi: 10.1146/annurev-ecolsys-110617-062453, PMID: 32733173 PMC7392196

[ref22] Ozaki S. Fritsch C. Valot B. Mora F. Cornier T. Scheifler R.. (2018). Does pollution influence small mammal diet in the field? A metabarcoding approach in a generalist consumer. Mol. Ecol. 27, 3700–3713. doi: 10.1111/mec.14823 30069953

[ref9002] Park J. K. Do Y. (2024). The difference and variation of gut bacterial community and host physiology can support adaptation during and after overwintering in frog population. Integr. Zool. 19, 631–645. doi: 10.1111/1749-4877.12798 38185804

[ref23] Parker B. J. Wearsch P. A. Veloo A. C. Rodriguez-Palacios A. (2020). The genus Alistipes: gut bacteria with emerging implications to inflammation, cancer, and mental health. Front. Immunol. 11:906. doi: 10.3389/fimmu.2020.00906, PMID: 32582143 PMC7296073

[ref25] Petit Bon M. Gunnarsdotter Inga K. Jónsdóttir I. S. Utsi T. A. Soininen E. M. Bråthen K. A. (2020). Interactions between winter and summer herbivory affect spatial and temporal plant nutrient dynamics in tundra grassland communities. Oikos 129, 1229–1242. doi: 10.1111/oik.07074

[ref26] Pinho C. J. Lopes E. P. Paupério J. Gomes I. Romeiras M. M. Vasconcelos R. (2022). Trust your guts? The effect of gut section on diet composition and impact of *Mus musculus* on islands using metabarcoding. Ecol. Evol. 12:e8638. doi: 10.1002/ece3.8638, PMID: 35309743 PMC8901889

[ref27] Rosshart S. P. Vassallo B. G. Angeletti D. Hutchinson D. S. Morgan A. P. Takeda K.. (2017). Wild mouse gut microbiota promotes host fitness and improves disease resistance. Cell 171, 1015–1028.e13. doi: 10.1016/j.cell.2017.09.016, PMID: 29056339 PMC6887100

[ref28] Sanders J. G. Powell S. Kronauer D. J. Vasconcelos H. L. Frederickson M. E. Pierce N. E. (2014). Stability and phylogenetic correlation in gut microbiota: lessons from ants and apes. Mol. Ecol. 23, 1268–1283. doi: 10.1111/mec.12611, PMID: 24304129

[ref29] Sato J. J. Shimada T. Kyogoku D. Komura T. Uemura S. Saitoh T.. (2018). Dietary niche partitioning between sympatric wood mouse species (Muridae: Apodemus) revealed by DNA meta-barcoding analysis. J. Mammal. 99, 952–964. doi: 10.1093/jmammal/gyy063

[ref30] Soininen E. M. Ehrich D. Lecomte N. Yoccoz N. G. Tarroux A. Berteaux D.. (2014). Sources of variation in small rodent trophic niche: new insights from DNA metabarcoding and stable isotope analysis. Isot. Environ. Health Stud. 50, 361–381. doi: 10.1080/10256016.2014.915824, PMID: 24830842

[ref31] Suzuki T. A. Phifer-Rixey M. Mack K. L. Sheehan M. J. Lin D. Bi K.. (2019). Host genetic determinants of the gut microbiota of wild mice. Mol. Ecol. 28, 3197–3207. doi: 10.1111/mec.15139, PMID: 31141224 PMC6660988

[ref9004] Tang L. Yan L. Jia H. Xiong Y. Ma X. Chu H.. (2023). Gut microbial community structure and function of Przewalski’ horses varied across reintroduced sites in China. Integr. Zool. 18, 1027–1040. doi: 10.1111/1749-4877.12699 36606497

[ref32] Ter Schure A. T. Pillai A. A. Thorbek L. Bhavani Shankar M. Puri R. Ravikanth G.. (2021). eDNA metabarcoding reveals dietary niche overlap among herbivores in an Indian wildlife sanctuary. Environ. DNA 3, 214–230. doi: 10.1002/edn3.161, PMID: 40260586

[ref33] Wang J. Laguardia A. Damerell P. J. Riordan P. Shi K. (2014). Dietary overlap of snow leopard and other carnivores in the Pamirs of northwestern China. Chin. Sci. Bull. 59, 3162–3168. doi: 10.1007/s11434-014-0370-y

[ref34] Wang Z. Zhang C. Li G. Yi X. (2022). The influence of species identity and geographic locations on gut microbiota of small rodents. Front. Microbiol. 13:983660. doi: 10.3389/fmicb.2022.983660, PMID: 36532505 PMC9751661

[ref35] Weinstein S. B. Martínez-Mota R. Stapleton T. E. Klure D. M. Greenhalgh R. Orr T. J.. (2021). Microbiome stability and structure is governed by host phylogeny over diet and geography in woodrats (*Neotoma spp.*). Proc. Natl. Acad. Sci. 118:e2108787118. doi: 10.1073/pnas.2108787118, PMID: 34799446 PMC8617456

[ref36] Weldon L. Abolins S. Lenzi L. Bourne C. Riley E. M. Viney M. (2015). The gut microbiota of wild mice. PLoS One 10:e0134643. doi: 10.1371/journal.pone.0134643, PMID: 26258484 PMC4530874

[ref37] Woo C. Kumari P. Eo K. Y. Lee W. S. Kimura J. Yamamoto N. (2022). Using DNA metabarcoding and a novel canid-specific blocking oligonucleotide to investigate the composition of animal diets of raccoon dogs (*Nyctereutes procyonoides*) inhabiting the waterside area in Korea. PLoS One 17:e0271118. doi: 10.1371/journal.pone.0271118, PMID: 35877678 PMC9312373

[ref38] Xiong M. Shao X. Long Y. Bu H. Zhang D. Wang D.. (2016). Molecular analysis of vertebrates and plants in scats of leopard cats (*Prionailurus bengalensis*) in Southwest China. J. Mammal. 97, 1054–1064. doi: 10.1093/jmammal/gyw061

[ref39] Xu P. Zhang X. F. Ma Y. (2021). Using 16S rDNA to study the diversity of the intestinalflora of the Oinghai Himalayan marmot. Chin. J. Zoonoses 37, 773–782. doi: 10.3969/j.issn.1001-6980.2021.09.004

[ref40] Zhang X. Y. Khakisahneh S. Liu W. Zhang X. Zhai W. Cheng J.. (2023). Phylogenetic signal in gut microbial community rather than in rodent metabolic traits. Nat. Sci. Rev. 10:nwad209. doi: 10.1093/nsr/nwad209, PMID: 37928774 PMC10625476

[ref41] Zhang X. Zou Y. Zou X. Xu Z. Nan X. Han C. (2022). DNA metabarcoding uncovers the diet of subterranean rodents in China. PLoS One 17:e0258078. doi: 10.1371/journal.pone.0258078, PMID: 35482781 PMC9049501

[ref9001] Zhao J. Feng T. An X. Chen X. Han N. Wang J.. (2024). Livestock grazing is associated with the gut microbiota and antibiotic resistance genes in sympatric plateau pika (Ochotona curzoniae). Integr. Zool. 19, 646–661. doi: 10.1111/1749-4877.12778 37828802

[ref42] Zhong W. Wang G. Zhou Q. Ma L. Wan X. Liu W. (2016). Spatial niche partitioning of coexisting small mammals in sand dunes. Ital. J. Zool. 83, 248–254. doi: 10.1080/11250003.2016.1139636

