# Supplementary material for: Associations between gut microbiota and diet composition of three arid-adapted rodent species from the Inner Mongolia grassland
Source: Front Microbiol. 2025 May 7;16:1569592. doi: 10.3389/fmicb.2025.1569592 (PMC12092225; doi:10.3389/fmicb.2025.1569592)
Supplement: Supplementary file 1 [file Table_1.doc]

Supplementary Material

| **Supplementary table 1.** Sampling Information   | ID | Rodent species | weight（g） | body length (mm) | tail length (mm) | hind foot length (mm) | ear length (mm) | gender | Collection site (°) | | --- | --- | --- | --- | --- | --- | --- | --- | --- | | 1 | *Spermophilus alashanicus* | 109.5 | 157 | 54 | 34 | 8 | ♀ | E105.795870 N38.846697 | | 2 | *S. alashanicuss* | 130 | 175 | 63 | 36 | 9 | ♀ | E105.795870 N38.846697 | | 3 | *S. alaschanicus* | 204 | 215 | 65 | 36 | 8 | ♂ | E105.795870 N38.846697 | | 4 | *S. alaschanicus* | 203 | 212 | 48 | 40 | 7 | ♂ | E105.795870 N38.846697 | | 5 | *S. alaschanicus* | 198 | 210 | 68 | 35 | 7 | ♂ | E105.795870 N38.846697 | | 6 | *S. alaschanicus* | 137 | 195 | 65 | 36 | 7 | ♀ | E105.795870 N38.846697 | | 7 | *S. alaschanicus* | 180 | 208 | 70 | 37 | 9 | ♀ | E105.795870 N38.846697 | | 8 | *S. alaschanicus* | 145 | 197 | 80 | 38 | 9 | ♀ | E105.795870 N38.846697 | | 1 | *S. dauricus* | 175 | 178 | 65 | 34 | 4 | ♀ | E119.952362 N43.670195 | | 2 | *S. dauricus* | 345 | 222 | 68 | 36 | 5 | ♀ | E119.952362 N43.670195 | | 3 | *S. dauricus* | 120 | 162 | 66 | 34 | 5 | ♀ | E119.952362 N43.670195 | | 4 | *S. dauricus* | 310 | 190 | 62 | 35 | 5 | ♀ | E119.952362 N43.670195 | | 5 | *S. dauricus* | 125 | 180 | 55 | 38 | 5 | ♀ | E119.952362 N43.670195 | | 6 | *S. dauricus* | 150 | 180 | 60 | 31 | 5 | ♂ | E119.952362 N43.670195 | | 7 | *S. dauricus* | 192 | 201 | 47 | 38 | 8 | ♂ | E119.952362 N43.670195 | | 8 | *S. dauricus* | 208 | 211 | 67 | 35 | 8 | ♂ | E119.952362 N43.670195 | | 9 | *S. dauricus* | 245 | 210 | 58 | 40 | 3 | ♂ | E117.766331 N48.966967 | | 10 | *S. dauricus* | 254 | 221 | 46 | 33 | 6 | ♂ | E117.766331 N48.966967 | | 11 | *S. dauricus* | 216 | 216 | 47 | 35 | 6 | ♂ | E117.766331 N48.966967 | | 12 | *S. dauricus* | 195 | 203 | 48 | 34 | 5 | ♀ | E117.766331 N48.966967 | | 13 | *S. dauricus* | 286 | 227 | 49 | 32 | 4 | ♂ | E117.766331 N48.966967 | | 14 | *S. dauricus* | 215 | 210 | 62 | 33 | 6 | ♀ | E117.766331 N48.966967 | | 15 | *S. dauricus* | 163 | 190 | 56 | 32 | 5 | ♂ | E117.766331 N48.966967 | | 16 | *S. dauricus* | 255 | 212 | 51 | 34 | 5 | ♀ | E117.766331 N48.966967 | | 1 | *Meriones unguiculatus* | 52 | 110 | 80 | 30 | 8 | ♀ | E119.386389 N42.799444 | | 2 | *M. unguiculatus* | 80 | 125 | 112 | 24 | 9 | ♂ | E119.386389 N42.799444 | | 3 | *M. unguiculatus* | 59 | 120 | 100 | 27 | 10 | ♀ | E119.386389 N42.799444 | | 4 | *M. unguiculatus* | 41 | 80 | 80 | 20 | 6 | ♀ | E119.386389 N42.799444 | | 5 | *M. unguiculatus* | 49 | 118 | 99 | 27 | 7 | ♂ | E120.527579 N43.927269 | | 6 | *M. unguiculatus* | 75 | 121 | 111 | 30 | 6 | ♂ | E120.527579 N43.927269 | | 7 | *M. unguiculatus* | 50 | 99 | 85 | 25 | 5 | ♂ | E120.527579 N43.927269 | | 8 | *M. unguiculatus* | 75 | 105 | 110 | 30 | 7 | ♀ | E120.527579 N43.927269 | | 9 | *M. unguiculatus* | 90 | 115 | 111 | 30 | 7 | ♀ | E120.527579 N43.927269 | | 10 | *M. unguiculatus* | 75 | 118 | 88 | 29 | 7 | ♂ | E120.527579 N43.927269 | | 11 | *M. unguiculatus* | 45 | 85 | 7 | 95 | 27 | ♂ | E120.527579 N43.927269 | | 12 | *M. unguiculatus* | 55 | 104 | 80 | 30 | 7 | ♂ | E120.527579 N43.927269 | |
| --- | --- | --- | --- | --- | --- | --- | --- | --- | --- | --- | --- | --- | --- | --- | --- | --- | --- | --- | --- | --- | --- | --- | --- | --- | --- | --- | --- | --- | --- | --- | --- | --- | --- | --- | --- | --- | --- | --- | --- | --- | --- | --- | --- | --- | --- | --- | --- | --- | --- | --- | --- | --- | --- | --- | --- | --- | --- | --- | --- | --- | --- | --- | --- | --- | --- | --- | --- | --- | --- | --- | --- | --- | --- | --- | --- | --- | --- | --- | --- | --- | --- | --- | --- | --- | --- | --- | --- | --- | --- | --- | --- | --- | --- | --- | --- | --- | --- | --- | --- | --- | --- | --- | --- | --- | --- | --- | --- | --- | --- | --- | --- | --- | --- | --- | --- | --- | --- | --- | --- | --- | --- | --- | --- | --- | --- | --- | --- | --- | --- | --- | --- | --- | --- | --- | --- | --- | --- | --- | --- | --- | --- | --- | --- | --- | --- | --- | --- | --- | --- | --- | --- | --- | --- | --- | --- | --- | --- | --- | --- | --- | --- | --- | --- | --- | --- | --- | --- | --- | --- | --- | --- | --- | --- | --- | --- | --- | --- | --- | --- | --- | --- | --- | --- | --- | --- | --- | --- | --- | --- | --- | --- | --- | --- | --- | --- | --- | --- | --- | --- | --- | --- | --- | --- | --- | --- | --- | --- | --- | --- | --- | --- | --- | --- | --- | --- | --- | --- | --- | --- | --- | --- | --- | --- | --- | --- | --- | --- | --- | --- | --- | --- | --- | --- | --- | --- | --- | --- | --- | --- | --- | --- | --- | --- | --- | --- | --- | --- | --- | --- | --- | --- | --- | --- | --- | --- | --- | --- | --- | --- | --- | --- | --- | --- | --- | --- | --- | --- | --- | --- | --- | --- | --- | --- | --- | --- | --- | --- | --- | --- | --- | --- | --- | --- | --- | --- | --- | --- | --- | --- | --- | --- | --- | --- | --- | --- | --- | --- | --- | --- | --- | --- | --- | --- | --- | --- | --- | --- | --- | --- | --- | --- | --- | --- | --- | --- | --- | --- | --- | --- | --- | --- | --- | --- | --- | --- | --- | --- | --- | --- | --- | --- | --- | --- |
